# Safety, tolerability, and efficacy estimate of evoked gamma oscillation in mild to moderate Alzheimer’s disease

**DOI:** 10.3389/fneur.2024.1343588

**Published:** 2024-03-06

**Authors:** Mihály Hajós, Alyssa Boasso, Evan Hempel, Monika Shpokayte, Alex Konisky, Chandran V. Seshagiri, Vitella Fomenko, Kim Kwan, Jessie Nicodemus-Johnson, Suzanne Hendrix, Brent Vaughan, Ralph Kern, Jonathan T. Megerian, Zach Malchano

**Affiliations:** ^1^Cognito Therapeutics, Inc., Cambridge, MA, United States; ^2^Department of Comparative Medicine, Yale University School of Medicine, New Haven, CT, United States; ^3^Pentara Corporation, Millcreek, UT, United States; ^4^Thompson Autism Center, CHOC Children’s, Orange, CA, United States

**Keywords:** Alzheimer’s disease, evoked gamma oscillation, OVERTURE clinical trial, brain atrophy, ADCS-ADL, MMSE

## Abstract

**Background:**

Alzheimer’s Disease (AD) is a multifactorial, progressive neurodegenerative disease that disrupts synaptic and neuronal activity and network oscillations. It is characterized by neuronal loss, brain atrophy and a decline in cognitive and functional abilities. Cognito’s Evoked Gamma Therapy System provides an innovative approach for AD by inducing EEG-verified gamma oscillations through sensory stimulation. Prior research has shown promising disease-modifying effects in experimental AD models. The present study (NCT03556280: OVERTURE) evaluated the feasibly, safety and efficacy of evoked gamma oscillation treatment using Cognito’s medical device (CogTx-001) in participants with mild to moderate AD.

**Methods:**

The present study was a randomized, double blind, sham-controlled, 6-months clinical trial in participants with mild to moderate AD. The trial enrolled 76 participants, aged 50 or older, who met the clinical criteria for AD with baseline MMSE scores between 14 and 26. Participants were randomly assigned 2:1 to receive self-administered daily, one-hour, therapy, evoking EEG-verified gamma oscillations or sham treatment. The CogTx-001 device was use at home with the help of a care partner, over 6 months. The primary outcome measures were safety, evaluated by physical and neurological exams and monthly assessments of adverse events (AEs) and MRI, and tolerability, measured by device use. Although the trial was not statistically powered to evaluate potential efficacy outcomes, primary and secondary clinical outcome measures included several cognitive and functional endpoints.

**Results:**

Total AEs were similar between groups, there were no unexpected serious treatment related AEs, and no serious treatment-emergent AEs that led to study discontinuation. MRI did not show Amyloid-Related Imaging Abnormalities (ARIA) in any study participant. High adherence rates (85–90%) were observed in sham and treatment participants. There was no statistical separation between active and sham arm participants in primary outcome measure of MADCOMS or secondary outcome measure of CDR-SB or ADAS-Cog14. However, some secondary outcome measures including ADCS-ADL, MMSE, and MRI whole brain volume demonstrated reduced progression in active compared to sham treated participants, that achieved nominal significance.

**Conclusion:**

Our results demonstrate that 1-h daily treatment with Cognito’s Evoked Gamma Therapy System (CogTx-001) was safe and well-tolerated and demonstrated potential clinical benefits in mild to moderate AD.

**Clinical Trial Registration:** www.ClinicalTrials.gov, identifier: NCT03556280.

## 1 Introduction

Alzheimer’s disease (AD) is a progressive neurodegenerative disease characterized by the gradual loss of cognitive and functional abilities, severe memory loss, and brain atrophy. Gamma oscillations, a 30–90 Hz voltage potential generated by synchronous neuronal activity is necessary for sensory processing, cognition, memory consolidation, which are greatly disrupted in AD (1–5). These disruptions along with network hypersynchrony and neuronal hyperexcitability have been shown to widely overlap with brain regions that develop pathological hallmarks and atrophy in AD patients (5–9). Accumulating evidence suggests that evoked 40 Hz gamma oscillation has the potential to alleviate AD pathology and preserve cognitive function in experimental transgenic animal models. Neuronal 40 Hz steady-state oscillations, evoked by daily optogenetic or sensory (visual and/or auditory) stimulation over several weeks have been shown to have multiple downstream biological effects, including attenuation of synaptic loss and neurodegeneration, reduction in amyloid and tau pathologies, and improvement in learning and memory in various AD mouse models (10–14). These original findings have been confirmed and extended by numerous subsequent publications (15–18), although not by all (19, 20).

Sensory-evoked steady state oscillations have been demonstrated in humans, and they attracted a substantial interest for their clinical applications (21–23). Sensory-evoked steady state oscillations are considered diagnostic tools, and indicators of disease progression (24–26). As a potential therapeutic intervention, sensory-evoked steady-state oscillations have been shown to facilitate long-term potentiation-like processes in the cortex and improve information processing and working memory in humans (27–31). Based on both preclinical and clinical findings summarized above, modulation of pathophysiological network activities by sensory evoked gamma oscillation may provide new strategies for disease-modifying treatments in AD.

Cognito Therapeutics, Inc. is developing an innovative medical device that elicits an EEG confirmed 40 Hz steady-state brain oscillations for at-home treatment of AD. Safety, tolerability, and potential effects on biomarkers of evoked gamma oscillation have been previously evaluated by two clinical trials, with 2–3 months of treatment (9, 32). In comparison, the present study is significantly larger and longer; it is a 2:1 randomized, sham-controlled, double-blind trial that evaluated the safety, tolerability, and estimated effect size of Cognito Therapeutics CogTx-001 medical device. Participants with mild to moderate AD received daily, 1-h treatment using the CogTx-001 medical device over a 6-month period. During the screening phase, stimulation intensities were tailored for each study participant in the clinical setting and the presence of treatment-evoked 40 Hz steady-state. EEG oscillations were verified for study inclusion.

Potential changes in cognition and function were evaluated by several clinical instruments, including the Alzheimer’s Disease Assessment Scale–Cognitive Subscale-14 (ADAS-Cog14), AD Composite Score (ADCOMS, and its version, MADCOMS tailored for mild to moderate AD participants), Clinical Dementia Rating (CDR), Alzheimer’s Disease Cooperative Study Activities of Daily Living (ADCS-ADL) and the Mini-Mental State Exam (MMSE). As an early phase clinical trial, without controlled clinical trial data to provide an accurate estimate of the expected treatment effect size, efficacy outcomes were considered exploratory. In addition to clinical outcomes, the trial measured changes in amyloid brain pathology using positron emission tomography (PET) analysis and brain atrophy using volumetric MRI. Results of the presented trial support a larger randomized, controlled clinical trial (HOPE, NCT05637801) to further explore the potential clinical benefits of Cognito Therapeutics CogTx-001 medical device in mild to moderate AD.

## 2 Methods

### 2.1 Clinical study participants

The trial enrolled participants of age 50 and older who met the National Institute on Aging–Alzheimer’s Association core clinical criteria for probable AD with MMSE scores between 14 and 26 and a clinical diagnosis of mild to moderate AD. Participants underwent MRI to exclude confounding pathologies, such as ischemic stroke, intracerebral macro-hemorrhages or more than 4 micro-hemorrhages, or any findings that would preclude accurate MRI and amyloid PET imaging. Additional exclusion criteria included: profound hearing or visual impairment; a history of seizures; and anti-epileptic treatment. A stable dose of cholinesterase inhibitors was permitted, however memantine use was excluded due to a potential inhibitory effect on gamma oscillations. Only participants with EEG-verified 40 Hz gamma oscillations in response to combined auditory–visual stimulation were randomized (see below). A reliable lead care partner was also required for enrollment. The main inclusion and exclusion criteria are listed in Supplementary Table S1. The trial was conducted over 5 clinical sites, including Boston Center for Memory, Newton, MA (PIs: Paul Solomon, Ph.D and Elizabeth Vassey, PsyD.) Brain Matters Research, Stuart, FL and Del Ray, FL (PI: Mark Brody M.D., CPI), The Cognitive and Research Center of New Jersey, Springfield, NJ (PI: Michelle Papka Ph.D.), and ActivMed Practices and Research, Methuen, MA (PI: Michael McCartney M.D.).

### 2.2 Optimization of sensory stimulation for safety and evoked gamma oscillation

Treatment was carried out with Cognito Therapeutics, Inc. CogTx-001 medical device that includes an eye-set for visual stimulation, headphones for auditory stimulation, and a handheld controller. During a clinical visit, the device was optimized for each participant’s tolerability to the stimuli, as well as the presence of EEG-verified 40 Hz steady-state gamma oscillations evoked by auditory and visual stimulation, at several different volumes and intensities, respectively (Supplementary Figure S1). Each stimulus level was presented continuously for 1–4 min. All EEG recordings were collected using a 64- or 32-channel ANT-Neuro eego systems (ANT Neuro b.v., Hengelo, Netherlands). Data were bandpass filtered and re-referenced to the common average of all EEG channels. Power spectral density (PSD) analysis of the responses to each stimulus level was used to estimate 40 Hz neural response. All EEG data analysis was conducted using MATLAB (The Mathworks, Inc. Natick, MA). Following the clinical visit used to optimize the treatment, each enrolled participant received their personalized device. During the therapy, participants could adjust stimulation intensities via the controller within a pre-set range of intensities that were shown to consistently evoke a gamma response.

### 2.3 Clinical study design

This was a multicenter, randomized 6-month, sham-controlled, double-blind clinical trial which took place in the United States (NCT03556280). Participants were assigned at a 2-to-1 ratio into active and sham arms. Participants in both arms used the same medical device and they followed the same treatment protocol, however the setting of stimulation parameters of the device was different between arms. The device was preset to evoke 40 Hz steady-state oscillation in active arm participants, whereas in sham arm participants preset stimulation did not evoke 40 Hz steady-state oscillation (due to on-going clinical trials, further details of sham stimulation parameters are currently withheld). Participants, study partners, and assessment raters were blinded to group assignment. The therapy was self-administered at home with the help of a care partner. Participants were required to use the device daily for an hour each day. Participants were advised to have the treatment in the morning hours, sitting comfortably, and to refrain from unnecessary movement during the stimulation or from falling asleep. The device captured information on day, time, and duration of usage, together with level of stimulation intensities; data was uploaded to a secured cloud server for remote monitoring. The 6-month randomized controlled trial phase was followed by a 12-month open label extension. The study was conducted in accordance with the Declaration of Helsinki and had ethics committee approval at each participating site. The CIP was developed in accordance with the requirements set forth in the United States Code of Federal Regulation, 21 CFR 812 Investigational Device Exemptions, ISO 14155:2011 Clinical Investigations of Medical Devices for Human Subjects, the Medical Device Directive 93/42/EEC of the European Union, and the Declaration of Helsinki by the World Health Organization (as amended in 2008). Institutional Review Board (IRB) review was carried out by Advarra, a full AAHRPP accredited research review service provider. Advarra’s IRB Organization (IORG) Number is 0000635 and IRB Registration number is 00000971. All participants provided written informed consent that adhered to all necessary policies.

### 2.4 Safety and tolerability endpoints

The primary study objective was to evaluate the safety and tolerability of the CogTx-001 medical device. Safety was evaluated by physical and neurological exams at baseline, 12-, and 24-weeks, monthly assessments of adverse events (AEs), and MRI. The incidence and nature of AEs were based on the safety population and coded using the Medical Dictionary for Regulatory Activities (MedDRA v16.1). Severity levels include mild, moderate, and severe. Therapy relationships were grouped into two categories: related and unrelated. Unrelated and unlikely were categorized as “unrelated.” Possible, probable, and definite were categorized as “related.” If a participant has the same AE on multiple occasions, the highest severity or therapy relationship recorded for the event will be presented. Tolerability was evaluated using adherence to treatment regime as measured by daily device use.

### 2.5 Clinical assessments and study endpoints

The present study evaluated several efficacy endpoints that have been widely used in AD clinical trials, including cognition and function as well as neuroimaging. The primary efficacy endpoint was the change in MADCOMs from baseline to 6 months. Secondary outcome measures included: changes from baseline in ADCS-ADL at 1, 2, 3, 4, 5, and 6 months, changes from baseline to 3 and 6 months in ADAS-Cog14, ADCOMS (a composite measure of 2 MMSE items and the 6 CDR items), Neuropsychiatric Inventory (NPI), CDR, MMSE, Columbia-Suicidality Severity Rating Scale (C-SSRS), Quality-of-Life Alzheimer’s Disease (QoL-AD), Zarit Burden Inventory (ZBI), PET amyloid, and volumetric MRI measurements.

### 2.6 MRI

Acquisition of 1.5 Tesla MRI at each imaging site followed a previously described standardized protocol (33) that was rigorously validated across different neuroimaging sites. MRI data was collected for safety and efficacy assessments. MRI scans were assessed for ARIA over the course of the study for safety by a neuroradiologist blinded to treatment assignment at a central reading facility (Imaging Corp). All 3D T1-weighted structural MRI images were processed by Biospective, Inc. (Montréal, QC H3B 2 T9, Canada) using their proprietary PIANO™ software. PIANO™ is a configurable, modular, pipeline-based system for fully automated processing of multi- modality images. PIANO™ was designed for high-throughput processing of large-scale, multi-center, neuroimaging data. PIANO™ was configured and validated specifically for this study to incorporate the study-specific details utilized by the core PIANO™ modules. For this study, a standard ADNI template and corresponding atlas was used. The regions of interest (ROIs) were defined on this anatomical template and included whole brain volume (cerebrum and cerebellum), including only brain parenchyma (not cerebrospinal fluid), whole brain cortex (cerebral cortical gray matter), lateral ventricle, occipital and temporal lobe volumes, and left and right hippocampal volumes. Cortical thickness was computed at each vertex as the distance between the inner and outer surfaces (34). Parametric maps were mapped to a standardized surface by non-rigid 2D surface registration to derive the spatially normalized measures (35). Cortical thickness values were generated for the following ROIs: composite temporal lobe (entorhinal, fusiform, inferior temporal gyrus, middle temporal regions) and occipital lobe (lingual, cuneus, superior occipital gyrus, inferior occipital gyrus, calcarine sulcus, middle occipital gyrus). All volumetric MRI analyses were controlled for total intracranial volume.

### 2.7 Amyloid-β PET imaging

Amyloid pathology was determined during enrolment, 3 and 6 months of treatment using Amyvid [18F] florbetapir PET imaging, performed 50–70 min following the intravenous administration of the tracer. PET images were obtained from the level of the vertex to the base of the skull. Axial, sagittal, and coronal views were provided and qualitatively classified as amyloid positive or negative based on the Amyvid prescribing pharmaceutical guidelines. For quantitative analysis, the amyloid PET images underwent several processing steps using Biospective’s PIANO™ software, including frame-to-frame motion correction, image smoothing, and co-registration to anatomical MRI. Following linear registration to the participant-specific, baseline T1-weighted, 3D anatomical MRI volume, the PET volumes were spatially normalized to reference space using the linear transformations derived from the anatomical MRI registration. ROI-based standardized uptake value ratio (SUVR) measures, generated by PIANO™, were obtained using automated atlas-based parcellation in stereotaxic space using the whole cerebellum and pons as reference regions. Composite ROI (frontal cortex, lateral temporal cortex, parietal cortex, somatosensory cortex, cuneus) values are presented here.

### 2.8 Statistical analysis

Statistical analyses were performed in SAS v9.4 or higher.

#### 2.8.1 Mild/moderate Alzheimer’s disease composite

The primary efficacy endpoint Mild and Moderate Alzheimer’s Disease Composite (MADCOMS) is a weighted composite based on components of ADAS-Cog 14, MMSE, and CDR-SB. MADCOMS was derived because ADAS-Cog14 is not specifically targeted to the mild/moderate stage of Alzheimer’s Disease necessitating a composite score optimized to the mild and moderate groups separately. The composite score is made of items derived from an iterative partial least squares regression analysis that uses time as the response variable and individual items of ADAS-Cog14, CDR-SB, and MMSE as the predictor/explanatory variables. Time was used as a surrogate for disease progression. Components included in the Mild and Moderate composites were selected by the iterative PLS regression. The analysis first excludes variables with a negative weighting, then excludes the variable with the lowest variable importance factor (VIP) below a set threshold of 0.5. Historic ADCS placebo data from the Simvastatin (LL), homocysteine (HC), Selegiline (SL), and NSAIDs (NS) were used to derive the weights. This process is repeated until the variables included in the model are of positive weight and have a VIP ≥ 0.5.

The composites derived from the PLS regression analysis are as follows:

*Moderate AD*: Comprehension*0.36390157 + Word Finding*0.10931155 + Ideational Praxis*0.42535667 + Naming Objects*0.65626894 + Word Recognition*0.05159097 + Word Recall*1.0698506 + Spoken Language*0.3019936 + Home and Hobbies*0.66529282 + Memory*0.12277257 - Orientation to Place*0.23001218 - Spell Backward*0.07980965 - Language and Praxis*0.18954955.

*Mild AD*: Word Finding*0.39065568 + Word Recall*1.14084544 + Spoken Language*1.09895590 + Personal Care*0.60865765 + Community Affairs*0.15706995 + Judgment*1.40920029 - Orientation to Time*0.27596627.

MADCOMS scores for individuals classified as mild at baseline were derived using the Mild AD equation. Individuals classified as having moderate AD at baseline were derived using the Moderate AD equation above. Individuals missing one or more of the items listed above were excluded.

#### 2.8.2 Primary efficacy analysis

The primary efficacy endpoint (MADCOMS) was analyzed in the intent to treat population based on treatment administered (ITT; received at least 1 day of therapy and underwent at least one post-baseline assessment) by comparing the change from baseline to 26 weeks of treatment using a linear mixed model with repeated measures. During the course of the study one individual was randomized incorrectly, ITT analyses are presented as treated in the main body of the manuscript to demonstrate the scope of treatment effects. The ITT analysis of individuals as randomized and associated counts can be found in the supplement (Supplementary Table S2). For both analyses, all available data was used. Three-month MMSE values were imputed for the primary efficacy variable (MADCOMS). MMSE values were imputed from baseline and 6-month values via straight line imputation. The remainder of missing data was not imputed but handled by the MMRM model (proc mixed) as performed by SAS v9.4. An unstructured covariance matrix was implemented for all analyses presented. Treatment group and time were included as fixed effects. Clinical site (covariance matrix variance components) and intercept were included as random effects. Age, baseline MMSE, and baseline efficacy parameter were used as covariates and treatment by time and treatment by baseline interactions were included in the model.

#### 2.8.3 Secondary efficacy analyses

Secondary endpoints included: ADAS-Cog 14, ADCOMS, NPI, CDR-SB, MMSE, ADCS-ADL, QoL-AD, ZBI, Change in Amyloid Burden (PET). All analyses except MMSE were analyzed using the model outlined for the primary efficacy analysis. PET SUVR analyses were adjusted for intercranial volume by including this as a covariate in the model. Volumetric brain MRI were analyzed as per the primary efficacy analysis with the exception that the model included intracranial volume as a baseline covariate.

MMSE was collected at baseline and the final 6-month visit. The change from baseline to 6 months was run as an ANCOVA, as implemented in the SAS v9.4 proc. mixed function. The model included age, baseline MMSE, and treatment as covariates. Site was included as a random effect (covariance matrix variance components). *Ad hoc* analysis included iADRS.

### 2.9 Summary statistics

Summary statistics of baseline data are presented as mean/median and standard deviation for continuous and count (percent) for discrete variables. Differences between active and sham groups were assessed using a t-test or chi-square for continuous or discrete variables, respectively.

## 3 Results

### 3.1 Patient demographic and baseline characteristics

**Figure 1:**
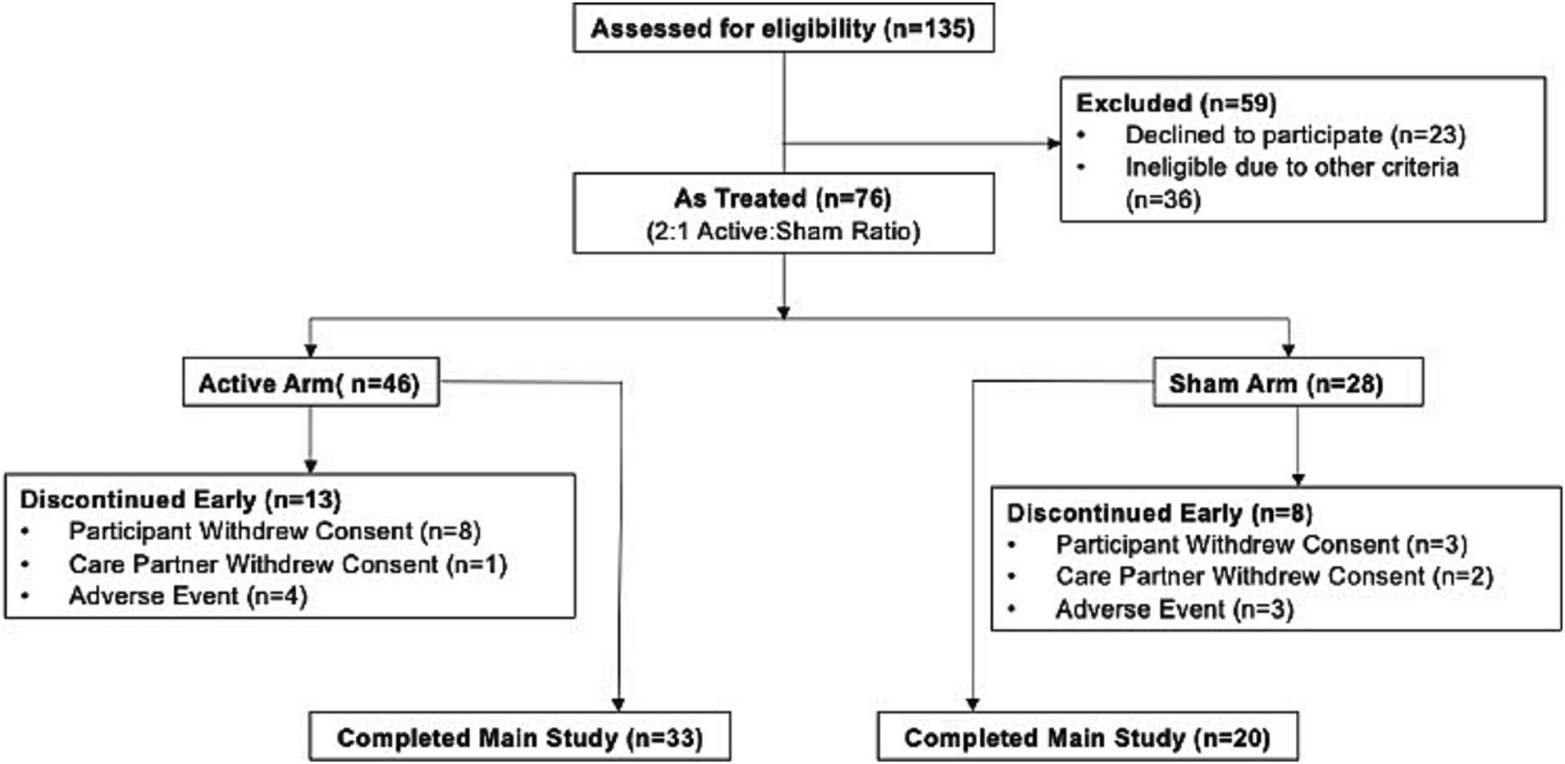
Patient allocation diagram. One participant that was randomly assigned to the sham group received active therapy during the trial and we therefore, hereafter, is included in the modified as-treated population. Details are provided in the methods. Inclusion and exclusion criteria can be found in the supplementary appendix (Supplementary Table S1).

Out of an initial cohort of 135 potential participants, 59 screen-failed after a thorough screening process. The primary reasons for screen failure were a lack consent to continuing participation (compromising 18.5% of the total screened individuals) and scores on the MMSE falling outside the predetermined range, affecting 7.4% of the screened group. Following the screening phase, 76 participants were enrolled into the trial with randomization taking place in a 2:1 ratio for treatment. There were 47 patients in the active group. and 29 in the sham group (Figure 1) and 74 received treatment (2 withdrew prior to treatment). The ITT population comprised 70 patients who received therapy and had at least one assessment post baseline (4 patients did not have a post baseline assessment). A total of 53 participants completed the trial, 33 (77%) in the active group and 20 (74%) in the sham group. The majority of early terminations were due to withdrawal of consent from participant or care partner. Seven participants (4 in active group and 3 in sham group) discontinued due to adverse events (AEs). Early termination rates were comparable between in the active (28%) and sham (29%) groups.

Demographic and baseline clinical characteristics of the participants are presented in Table 1. Imbalances between active and sham group participants at baseline were observed in age, ADAS-Cog14, MMSE and CDR scores. These imbalances were accounted for in the statistical analysis. There were no significant differences between active and sham group participants in MADCOMS, ADCS-ADL, Aβ-PET SUVR, MRI whole brain volume, or distribution of participants classified as Aβ positive or negative, based on 1.12 cut-off composite SUVR values. Since diagnosis for enrolment was based on a clinical diagnosis of AD, amyloid positive (*n* = 50, 71%) and negative (*n* = 20, 28%) participants were enrolled in the trial.

**Table 1:** Demographics and baseline characteristics.

	As treated				As randomized			
Characteristic	Sham	Active	Overall	*P*-value	Sham	Active	Overall	*P*-value
Age								
N	27	43	70	0.0092	28	42	70	0.0133
Mean (SD)	75.6 (10.04)	69.7 (7.99)	72.0 (9.21)		75.3 (9.95)	69.8 (8.08)	72.0 (9.21)	
Race *n* (%)								
Other		1 (2.3%)	1 (1.4%)	0.4248		1 (2.4%)	1 (1.4%)	0.4109
White	27 (100.0%)	42 (97.7%)	69 (98.6%)		28 (100.0%)	41 (97.6%)	69 (98.6%)	
Sex *n* (%)								
Female	12 (44.4%)	24 (55.8%)	36 (51.4%)	0.3542	13 (46.4%)	23 (54.8%)	36 (51.4%)	0.4943
Male	15 (55.6%)	19 (44.2%)	34 (48.6%)		15 (53.6%)	19 (45.2%)	34 (48.6%)	
APOE4 status *n* (%)								
Heterozygous	10 (37.0%)	16 (37.2%)	26 (37.1%)	0.594	10 (35.7%)	16 (38.1%)	26 (37.1%)	0.3664
Homozygous	2 (7.4%)	3 (7.0%)	5 (7.1%)		2 (7.1%)	3 (7.1%)	5 (7.1%)	
Carrier		3 (7.0%)	3 (4.3%)		16 (57.1%)	19 (45.2%)	35 (50.0%)	
Non-carrier	15 (55.6%)	20 (46.5%)	35 (50.0%)			4 (9.5%)	4 (5.7%)	
Unknown		1 (2.3%)	1 (1.4%)					
PET SUVR status (cerebellum reference region; 1.12 cutoff) *n* (%)								
Negative	9 (33.3%)	11 (25.6%)	20 (28.6%)	0.4847	9 (32.1%)	11 (26.2%)	20 (28.6%)	0.5892
Positive	18 (66.7%)	32 (74.4%)	50 (71.4%)		19 (67.9%)	31 (73.8%)	50 (71.4%)	
Cerebellum SUVR composite								
N	18	32	50	0.5734	19	31	50	0.9642
Mean (SD)	1.4 (0.21)	1.4 (0.15)	1.4 (0.17)		1.4 (0.22)	1.4 (0.14)	1.4 (0.17)	
AD quality of life score—family report								
N	27	43	70	0.4444	28	42	70	0.6817
Mean (SD)	36.8 (6.58)	37.9 (5.45)	37.5 (5.89)		37.1 (6.68)	37.7 (5.37)	37.5 (5.89)	
AD quality of life score—self report								
N	27	43	70	0.3589	28	42	70	0.429
Mean (SD)	39.9 (5.91)	41.0 (4.27)	40.5 (4.95)		40.0 (5.83)	40.9 (4.31)	40.5 (4.95)	
ADAS-Cog 11 total score								
N	26	43	69	0.0219	27	42	69	0.0309
Mean (SD)	22.1 (10.91)	17.2 (6.23)	19.1 (8.56)		21.8 (10.78)	17.3 (6.29)	19.1 (8.56)	
ADAS-Cog 14 total score								
N	26	43	69	0.0102	27	42	69	0.0164
Mean (SD)	34.8 (13.03)	27.9 (8.58)	30.5 (10.92)		34.4 (12.94)	28.0 (8.67)	30.5 (10.92)	
ADCOMS								
N	26	43	69	0.0079	27	42	69	0.0162
Mean (SD)	0.8 (0.34)	0.6 (0.21)	0.6 (0.28)		0.7 (0.34)	0.6 (0.21)	0.6 (0.28)	
Basic ADCS-ADL score								
N	27	43	70	0.6483	28	42	70	0.7429
Mean (SD)	21.2 (1.28)	21.4 (1.36)	21.3 (1.32)		21.3 (1.27)	21.4 (1.38)	21.3 (1.32)	
Instrumental ADCS-ADL score								
N	26	43	69	0.3394	27	42	69	0.4271
Mean (SD)	42.0 (9.91)	44.2 (8.35)	43.4 (8.96)		42.3 (9.81)	44.1 (8.42)	43.4 (8.96)	
Total ADCS-ADL score								
N	26	37	63	0.1398	27	36	63	0.1876
Mean (SD)	63.3 (10.65)	66.9 (8.54)	65.4 (9.56)		63.6 (10.55)	66.8 (8.64)	65.4 (9.56)	
CDR global								
N	27	43	70	<0.0001	28	42	70	0.0003
Mean (SD)	1.1 (0.53)	0.7 (0.25)	0.9 (0.42)		1.1 (0.53)	0.7 (0.25)	0.9 (0.42)	
CDR sum of boxes								
N	27	43	70	0.0018	28	42	70	0.0046
Mean (SD)	6.2 (2.79)	4.6 (1.54)	5.2 (2.25)		6.1 (2.81)	4.6 (1.54)	5.2 (2.25)	
MMSE-total score								
N	27	43	70	0.0477	28	42	70	0.0817
Mean (SD)	19.7 (3.85)	21.4 (3.28)	20.8 (3.59)		19.9 (3.87)	21.4 (3.30)	20.8 (3.59)	
Mild/Moderate ADCOMS (MADCOMS)								
N	26	43	69	0.3255	27	42	69	0.431
Mean (SD)	7.6 (2.63)	7.0 (1.96)	7.2 (2.23)		7.5 (2.61)	7.0 (1.97)	7.2 (2.23)	
NPI total score								
N	26	36	62	0.2333	26	36	62	0.2333
Mean (SD)	3.4 (3.91)	4.5 (3.36)	4.0 (3.61)		3.4 (3.91)	4.5 (3.36)	4.0 (3.61)	
Whole brain volume in cm^3^								
N	20	36	56	0.2302	21	35	56	0.3179
Mean (SD)	1017.7 (122.80)	1060.7 (129.35)	1045.4 (127.63)		1023.2 (122.27)	1058.7 (130.64)	1045.4 (127.63)	
ZBI total score								
N	27	43	70	0.7900	28	42	70	0.5678
Mean (SD)	21.9 (14.81)	22.8 (13.28)	22.5 (13.79)		21.3 (14.88)	23.3 (13.14)	22.5 (13.79)	

As Treated and As Pre-Specified populations; PET, positron emission tomography; SUVR, standardized uptake value ratio; CDR-SB, clinical dementia rating-sum of boxes; NPI total score, Neuropsychiatric Inventory Score; ZBI total score, Zarit Burden Interview Score.

### 3.2 Concomitant medications

The most frequent concomitant medication used in the trial was donepezil (*n* = 47, 64% of all participants, taking a fixed dose over the trial); donepezil use was higher in the sham group (*n* = 21, 75%) than the active group (*n* = 26, 57%) participants.

### 3.3 Safety and tolerability

The safety population included all trial participants who received at least one treatment (*n* = 74; 46 active; 28 sham). There were no reported unanticipated AEs due to the device over a 6-month treatment period. Treatment-emergent AEs (TEAEs) and treatment-related AEs (TRAEs) are shown in Table 2. Among AEs, musculoskeletal and connective tissue disorders included muscle spasms, arthralgia, back pain, cervical spinal stenosis, and neck pain. Nervous systems disorders included headache, agitation, dizziness, balance disorder, head discomfort, speech disorder, disorientation, gait disturbance, insomnia, migraine, sleep paralysis, and syncope. TEAEs were similar in active (*n* = 30, 65%) and sham (*n* = 20, 71%) groups. TRAEs were reported in 17 (35%) of active group participants and in 7 (25%) of sham group participants. Among the TRAEs (defined as possibly, probably, or definitely related to treatment/device by the site principal investigator), all were mild except for 3 events; 2 moderately severe events (tinnitus, active participant and agitation, sham participant) and 1 severe event (acute confusional state, active participant) were reported. Tinnitus was reported by 7 (15%) of active group participants and was not reported in the sham group, headache was reported by 10 (22%) of active group participants and by 3 (11%) of sham group participants. TRAEs (eye pain and disorientation) reported in the sham group occurred at low frequency (2 participants, 7%). C-SSRS showed no observed increase in suicidality in the active compared to the sham group. No deaths were reported during the study. Analysis of MRI data, reviewed by a neuroradiologist blinded at treatment arms at a central reading facility (Biospective Imaging Corp), demonstrated that no vasogenic edema and sulcal effusions (ARIA-E) or hemosiderin deposits (ARIA-H) greater than 10 mm were present in the participants who had 6-month magnetic resonance imaging (*n* = 52).

**Table 2:** Treatment emergent adverse events and treatment related adverse events.

Treatment emergent adverse events (TEAE)	Active	Sham	Treatment related adverse events (TREA)	Active	Sham
System order class	*N* = 46	*N* = 28	System order class	*N* = 46	*N* = 28
Preferred term	n (%)	n (%)		n (%)	n (%)
Number of subjects with any TEAE	30 (65.2)	20 (71.4)	Headache	10 (21.7)	3 (10.7)
Ear and labyrinth disorders	9 (19.6)	0 (0.0)	Tinnitus	7 (15.2)	0 (0.0)
Tinnitus	7 (15.2)	0 (0.0)	Eye pain	0 (0.0)	2 (7.1)
Eye disorders	3 (6.5)	3 (10.7)	Disorientation	0 (0.0)	2 (7.1)
Eye pain	0 (0.0)	2 (7.1)			
Gastrointestinal disorders	3 (6.5)	3 (10.7)			
General disorders and administration site conditions	2 (4.3)	4 (14.3)			
Oedema	0 (0.0)	2 (7.1)			
Infections and infestations	7 (15.2)	7 (25.0)			
Nasopharyngitis	0 (0.0)	6 (21.4)			
Injury, poisoning and procedural complications	2 (4.3)	2 (7.1)			
Skin abrasion	0 (0.0)	4 (14.3)			
Investigations	3 (6.5)	0 (0.0)			
Musculoskeletal and connective tissue disorders	6 (13.0)	1 (3.6)			
Nervous system disorders	14 (30.4)	10 (35.7)			
Headache	10 (21.7)	4 (14.3)			
Agitation	2 (4.3)	2 (7.1)			
Dizziness	2 (4.3)	2 (7.1)			
Disorientation	0 (0.0)	2 (7.1)			
Psychiatric disorders	7 (15.2)	4 (14.3)			
Anxiety	1 (2.2)	2 (7.1)			
Restlessness	0 (0.0)	2 (7.1)			
Respiratory, thoracic and mediastinal disorders	1 (2.2)	2 (7.1)			
Skin and subcutaneous tissue disorders	4 (8.7)	0 (0.0)			

Device-recorded usage throughout the treatment period demonstrated high adherence and tolerability to daily therapy (calculated as the percentage of days in the study where the device was used for 45 min or more). The average adherence across all participants who completed the main study was 85.1%. Among completers, the adherence rates were not statistically significant between active vs. sham participants, although sham participants had slightly higher average adherence rate (active: 81.3% vs. sham: 92.1%, *p* = 0.063, Kolmogorov–Smirnov test). Participants who terminated early were similar (28%) in both arms, and this group showed similar adherence rates of 77% in active and 74% in sham participants. Furthermore, the majority of the RCT completers (83%) entered the open label extension phase of the trial (36).

### 3.4 Effectiveness of patient blinding

The majority (85% participants, 76% care partners, 97% clinical raters) reported being unsure of treatment assignment. Of the participants that responded to the blinding assessment at their end of study visit and who indicated that they were somewhat or completely confident of treatment assignment, there was no differences between the active and sham groups in identification of the correct assignment across participants and clinical raters. Among study completers, care partners correctly identified active treatment assignment (relative to confidence in receiving the sham) only 50% of the time. Therefore, there was no evidence of unblinding in participants, care partners or clinical raters.

### 3.5 Effects on clinical measures

#### 3.5.1 Primary outcomes

**Figure 2:**
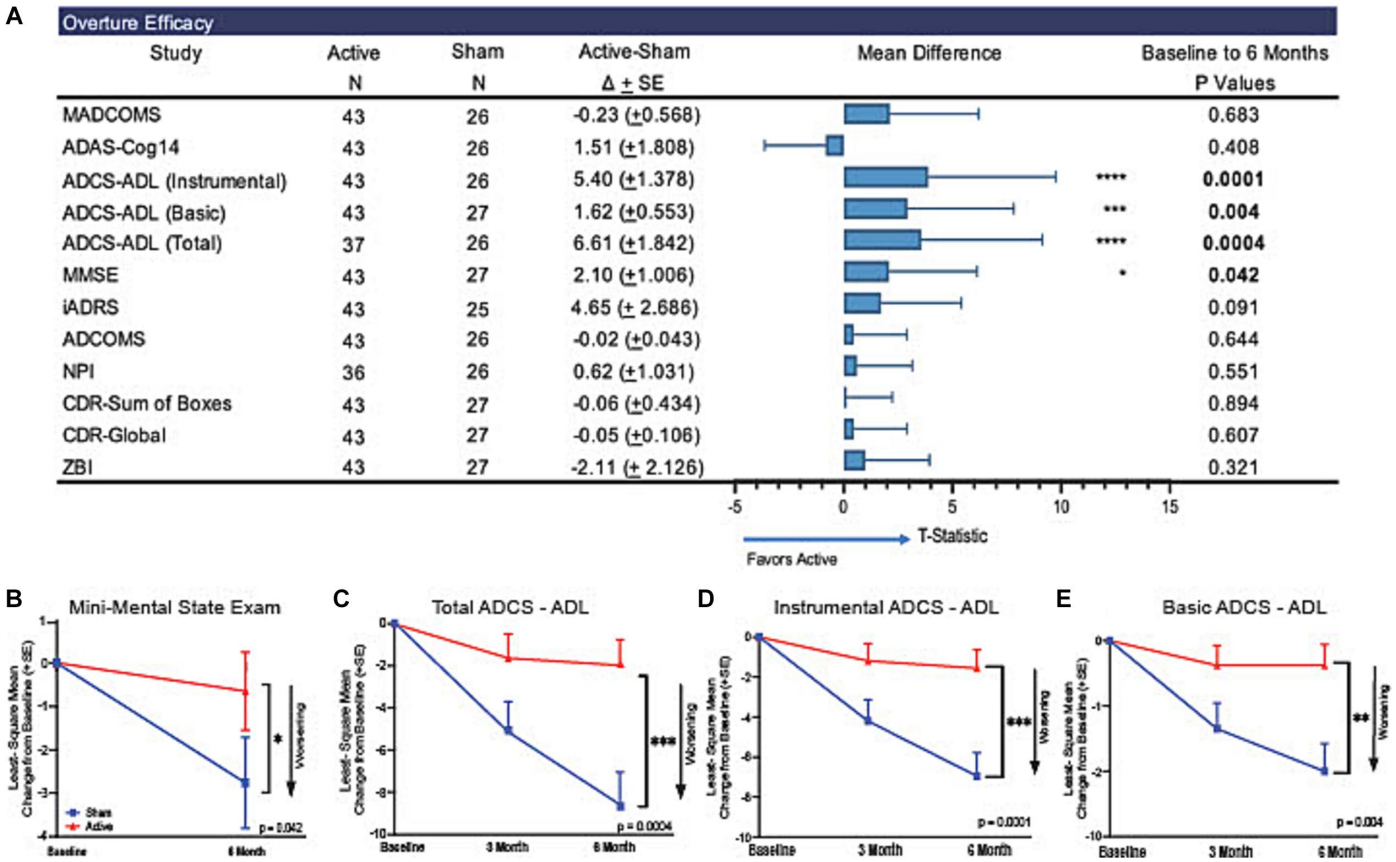
Trajectory of functional and cognitive abilities across 6 months of treatment. In the present trial participants that received active treatment demonstrated slower cognitive decline on the MMSE and ADCS-ADL over the course of 6 months of treatment compared to participants that received sham treatment. **(A)** Forest plot of LSMeans (SD) of efficacy outcomes to explore the association between the sham and active arms for each subgroup. Statistical values are represented in the table (N, LSMeans, and *P*-values). There was a significant difference between the active and sham groups trajectories as measured by the **(B)** MMSE (*p* = 0.042). Significant differences between active and sham arms at the end of the 6-month trial can be observed across scores on the ADCS-ADL **(C)** total (*p* = 0.0004), **(D)** basic (*p* = 0.004), and **(E)** instrumental (*p* = 0.0001). Functional abilities in the active group were retained during the 6 months of treatment compared to significant worsening in the sham group. *p < 0.05, **p < 0.005, ***p < 0.0005. Arrows point in the direction of worsening scores.

The primary outcome measure, MADCOMS, did not show separation between active and sham groups assessed as changes from baseline to 6 months (ΔLS Mean [SE]: −0.23 (0.568); 95% CI of difference: [−1.38, 0.91], *p* = 0.6825, Figure 2A; Supplementary Figure S2A).

#### 3.5.2 Secondary outcomes

These analyses are considered descriptive, exploratory assessments since prespecified primary outcome measure was not met, and multiple comparison was not carried out. MMSE showed a 76% lower decline from baseline in the active treatment group compared to the sham group that was nominally significant (ΔLS Mean [SE]: 2.10 (1.006), 95% CI of difference: [0.08, 4.12], *p* = 0.0417, Figure 2B). Similarly, the ADCS-ADL total score showed a 77% lower decline from baseline in the active treatment group compared to the sham group that was nominally significant (ΔLS Mean [SE] = 6.61 (1.842); 95% CI of difference [2.98, 10.23], *p* = 0.0004, Figure 2C). We observed a lower decline in the active treatment group compared to the sham group in the instrumental ADCS-ADL score (ΔLS Mean [SE]: 5.40 (1.378); 95% CI of difference: [2.69, 8.11], *p* = 0.0001, Figure 2D) and in the basic ADCS-ADL score (ΔLS Mean [SE]: 1.62 (0.553), 95% CI of difference: [0.53, 2.71] *p* = 0.004, Figure 2E) both were nominally significant.

The ADAS-Cog14 did not show differences between the active treatment group and the sham group at 6 months (ΔLS Mean [SE]: 1.51 (1.808); 95% CI of difference: [−2.13, 5.15], *p* = 0.4083, Supplementary Figure S2B). CDR-SB scores were not different between sham and active arms at 6 months (ΔLS Mean [SE]: −0.06 (0.434); 95% CI of difference: [−0.93, 0.82], *p* = 0.8941, Supplementary Figure S2C). No treatment differences were observed in ADCOMS, NPI, QoL and ZBI. These additional clinical outcomes are described in Supplementary Table S2.

The Integrated Alzheimer’s Disease Rating Scale (iADRS) was evaluated as an exploratory outcome measure, with higher scores indicating greater function and cognition. Although iADRS scores showed a trend favoring the treatment group, the difference in LS means between sham and treatment groups did not reach nominal statistical significance (ΔLS Mean [SE]: 4.65 (2.686), 95% CI of difference: [−0.76, 10.06], *p* = 0.0906, Supplementary Table S2).

### 3.6 Amyloid PET imaging

**Figure 3:**
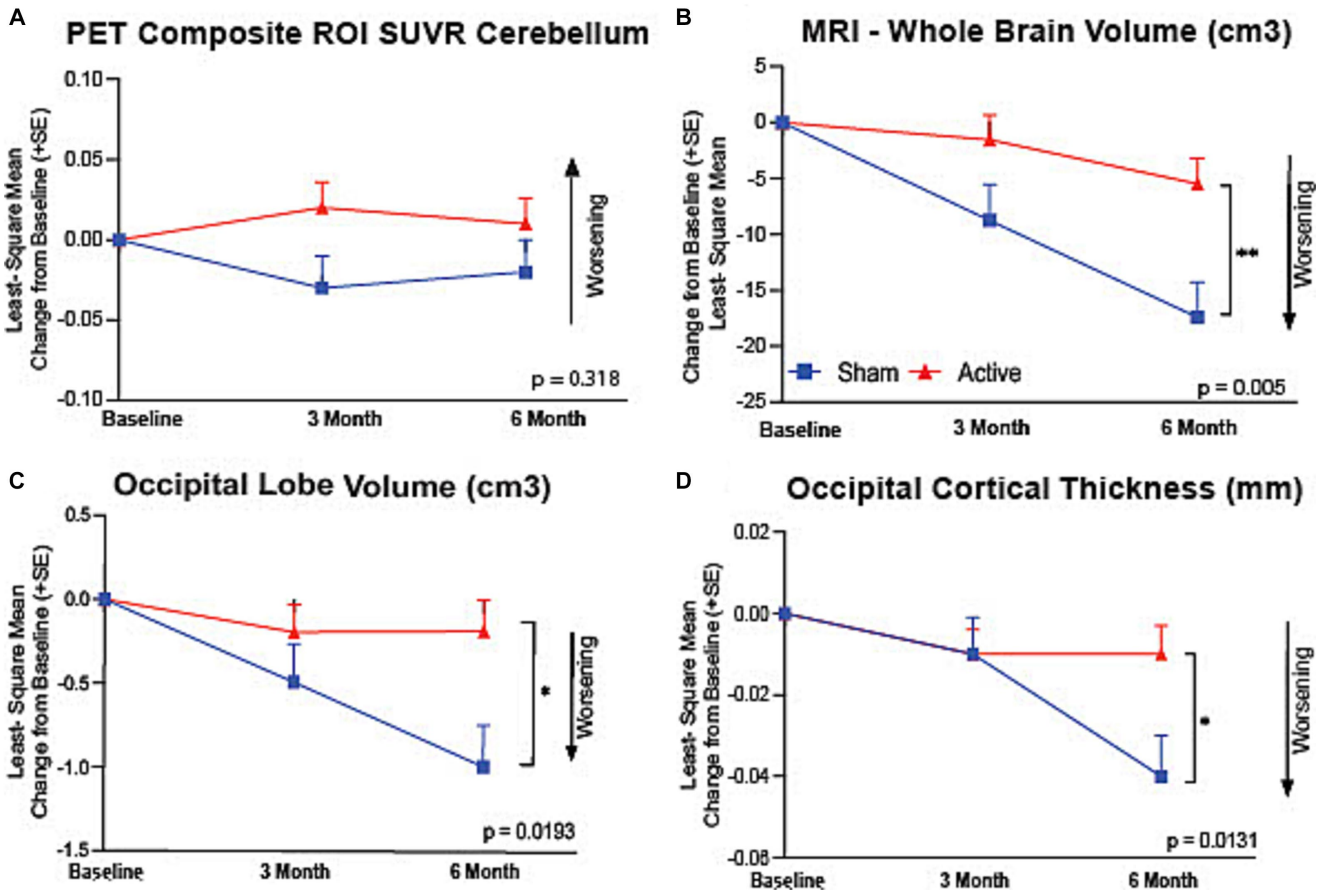
Preservation of brain volume assessed by vMRI. **(A)** There was no significant difference between active and sham groups in their PET Composite ROI SUVR referenced to cerebellum (*p* = 0.318). Active arm participants showed a significantly reduced volumetric change in **(B)** whole brain volume (*p* = 0.005), **(C)** occipital lobe volume (*p* = 0.019), and **(D)** occipital cortical thickness (*p* = 0.013) assessed by MRI at baseline and at 3- and 6-months of the therapy. *p < 0.05, **p < 0.005.

Eleven out of 43 (25.6%) active participants and 9 out of 27 (33.3%) had amyloid PET SUVR values below the 1.12 cutoff composite value, using the cerebellum as a reference region. There was no significant difference in mean PET SUVR baseline values between active treatment group (1.3 ± 0.23, *n* = 39) and the sham group (1.2 ± 0.25, *n* = 23), or at 6 months (active treatment group: 1.4 ± 0.22, *n* = 27; sham group; 1.3 ± 0.22, *n* = 18), including both amyloid positive and negative participants (Figure 3A). Similarly, no difference in 6-month PET outcomes were observed including only participants with baseline PET SUVR values >1.12 at baseline (active group baseline: 1.4 ± 0.16, *n* = 29; 6-month: 1.4 ± 0.16, *n* = 23; sham group: baseline: 1.4 ± 0.20, *n* = 15, 6-month: 1.4 ± 0.15, *n* = 13). Furthermore, no differences seen in individual brain region values between active and sham arm participants (data not shown).

### 3.7 Brain volumetric MRI

We observed a 69% reduction in whole brain volume loss in the active treatment group compared to the sham group at 6 months (ΔLS Mean [SE]: 11.93 (3.97) cm^3^, 95% CI of difference: [3.87, 19.99], *p* = 0.0049, Figure 3B) that was nominally significant. The active treatment group compared to the sham group also showed a reduced loss of occipital lobe volume (ΔLS Mean [SE]: 0.03 (0.01) mm, 95% CI of difference: [0.008, 0.059], *p* = 0.0131) (Figure 3C) and occipital cortical thickness (ΔLS Mean [SE]: 0.81 (0.32) cm^3^, 95% CI of difference: [0.01, 1.48], *p* = 0.0193, Figure 3D), that were nominally significant. Hippocampal volume changes were not different between the active treatment group and the sham group. Brain morphological changes were further analyzed by exploring correlations between distinct brain regions, including a previously established inverse correlation between whole brain volume loss and lateral ventricle expansion. In active arm participants, a significant (*p* = 0.0001) inverse correlation was found in volumetric changes between whole brain and lateral ventricles. In the sham group, additional correlations were established between volumetric changes in brain regions that are known to be connected to AD disease progression (37), including whole brain and temporal cortical lobe volumes (*r* = 0.85, *p* = 0.01) (Supplementary Figures S3A,B).

### 3.8 Correlations between brain volumetric MRI and clinical endpoints

Correlations between changes in brain volumetric and clinical endpoints were also established. In active arm participants, correlations were seen between changes in occipital lobe volume and ADCS-ADL total (*r* = 0.78, *p* = 0.02) and ADCS-ADL instrumental (*r* = 0.82, *p* = 0.01) scores, a relationship previously reported in AD patients (37). Additional correlations between volumetric and clinical changes are displayed on Supplementary Figures S3C,D; note that treatment modified correlation seen in the sham group participant.

### 3.9 Clinical outcomes stratified by baseline PET amyloid status

ADCS-ADL total score decline was reduced in the active treatment group compared to the sham group in amyloid PET positive participants (ΔLS Mean [SE]: 4.96 (2.113), 95% CI of difference: [0.80, 9.12], *p* = 0.0197) and in the amyloid PET negative participants (ΔLS Mean [SE]: 10.53 (3.349), 95% CI of difference: [3.93, 17.12], *p* = 0.0019) (Supplementary Figures S4A,B), that was nominally significant. Similar MMSE and whole brain volume trends were observed in amyloid PET positive and negative participants (Supplementary Table S3; Supplementary Figures S4C,B).

## 4 Discussion

The present clinical trial demonstrated that one-hour, daily treatment using Cognito’s Evoked Gamma Therapy System (CogTx-001) over a six-month period was safe, well tolerated, and showed high treatment adherence in participants with mild to moderate AD. Although a treatment difference was not demonstrated between the active treatment group and the sham group in MADCOMS, the primary efficacy outcome measure, nominally significant slowing of cognitive and functional decline was demonstrated in both MMSE and ADCS-ADL. Other secondary and exploratory outcomes did not demonstrate treatment differences. Furthermore, concordant reduction of MRI whole brain atrophy was demonstrated and important MRI correlations with clinical outcomes were observed. PET amyloid SUVR did not show treatment effects between the active treatment group and the sham group at 6 months.

This study achieved the primary objectives of safety, tolerability, and feasibility. The safety profile observed expands on observations in two previous clinical studies that also demonstrated good safety and tolerability: a delayed start, 4- or 8-weeks trial in 10 participants with mild cognitive impairment (32), and a single-blinded, randomized, placebo-controlled 3- months, pilot trial in 15 participants with mild probable AD dementia (9). ARIA is an important safety consideration in AD clinical trials; we did not observe MRI findings of ARIA-E or ARIA-H in any study participant. While treatment emergent adverse events occurred at a similar frequency in the active treatment and sham groups, we observed a higher occurrence of headache and tinnitus. A similar proportion of active treatment and sham treatment participants discontinued due to AEs. Treatment tolerability was demonstrated by device usage data, which showed a high adherence in the active treatment and sham groups. Furthermore, the retention rate of the current trial (early termination rate of 28 and 29%) was comparable to those of previous clinical trials which enrolled mild- to moderate AD participants (early termination of 11–37%, average of 23%), and being similar in both active and placebo groups (38). Tolerability to treatment was also indicated by the high rate (83%) of participants entering the open label extension phase of the trial (36). These observations contribute to growing clinical trial experience that confirms the safety and tolerability of Cognito’s Evoked Gamma Therapy System (CogTx-001) in mild to moderate AD. Effective blinding in participants, care partners and raters were confirmed throughout the study, and was consistently maintained in study completers and non-completers.

Although MADCOMES, the primary outcome measure did not separate treatment groups, descriptive analysis of secondar outcome measures evaluated potential effects on cognitive and functional abilities of active group participants. A previous study that evaluated the effect of 3 months of 1-h daily 40 Hz auditory and visual stimulation reported improvement in a face-name associative memory test in mild AD (9). While we observed a 76% reduction in MMSE decline and 77% reduction in ADCS-ADL decline in the active treatment group compared to the sham group at 6 months in this study, ADAS-Cog14 did not demonstrate a significant treatment effect. Although ADAS-Cog14 and MMSE values show significant correlations, the reasons for the apparent discrepancy between MMSE and ADAS-Cog14 results are unclear. MMSE is the most frequently used cognitive screening instrument in clinical practice. Historically the ADAS-Cog14 has been considered a sensitive efficacy measure in the evaluation of cholinesterase inhibitors (39), and therefore might be particularly responsive to cognitive function closely related to cholinergic neurotransmission, including hippocampal memory circuits. A non-significant reduction in decline in iADRS in active compared to sham participants was observed.

We detected nominally significant stabilization of daily function following treatment in ADCS-ADL total, instrumental and basic scores. These results confirm our previous findings showing improved sleep and reduced ADCS-ADL decline in a subpopulation of OVERTURE trial participants (40). These findings indicate a potential, broad beneficial effect of the treatment on multiple functional domains (41). Clinicians weigh heavily on patient function in the distinction between AD-MCI and AD-dementia. These declines have the greatest impact to the AD patient and result in progressive loss of independence, need for caregiver assistance, and eventually in assisted living. AD patients and their family members often seek medical evaluation when functional decline is observed following an initial period of cognitive decline (42).

Enrolment of participants in this clinical trial was based on a clinical diagnosis of AD. The amyloid status of participants was determined at baseline and potential changes in amyloid burden were additionally assessed by amyloid PET imaging during the treatment. Most experimental studies demonstrated that 40 Hz sensory stimulation reduced amyloid Aβ_40_ and Aβ_42_ concentrations in homogenized brain tissue and amyloid plaques assessed by immunocytochemical method in transgenic mice (13–18) [but see also (19, 20)]. The present study did not demonstrate a reduction in amyloid plaques as measured by PET SUVR. This finding is in line with previous reports, including no change in PiB SUVR values after 10 days, daily 40 Hz light stimulation in AD participants (43), or unchanged Aβ_42_ CSF concentrations after 8 weeks of auditory/visual sensory stimulation in amyloid positive MCI participants (32). Nevertheless, these results do not exclude the possibility that sensory-evoked gamma oscillation could impact brain soluble amyloid species in AD patients which cannot be detected by PET signals. Follow up studies, including measurements of various cerebrospinal fluid biomarkers will contribute to our understanding of how evoked neuronal gamma oscillations could differentially impact amyloid pathology in experimental studies and in AD patients. Nonetheless, it can be stated that the currently observed clinical benefits of the treatment are not mediated by reduction in amyloid plaque load, and the clinical benefits were equally demonstrated in PET amyloid positive and negative participants. These findings also indicate a potential treatment option for patients who are diagnosed with AD on clinical presentation without PET confirmation of amyloid positivity, which is a considerable segment of the dementia population (44).

Neuronal loss is the fundamental pathology in neurodegenerative diseases, including AD. Using MRI techniques, neuronal loss can be monitored as brain atrophy assessed by measurements of changes in volumes of the whole brain or its subregions such as the lateral ventricle, cortical and hippocampal volumes (45). Structural MRI measures also effectively indicate rates of brain atrophy, which are considered useful in tracking disease progression and as a potential outcome measure for clinical trials (46). The most intriguing result of the present trial was the observed 69% reduction in brain volume loss in the active treatment group compared to the sham group. Annualized whole brain atrophy rate in sham group participants (2.77% per year) was comparable to previously reported as annualized brain atrophy rate (2.34 and 2.40% per year) in mild to moderate AD patients (47, 48). Reduction in whole brain volume loss showed a strong inverse correlation with lateral ventricle enlargement and was also consistent with our clinical observations (34). Furthermore, analysis of the occipital lobe, where the most profound sensory-evoked gamma oscillations are observed (49), a significant reduction in loss of occipital lobe volume and cortical thickness were demonstrated. It is known that white matter volume loss and demyelination also contribute to brain atrophy in AD (50, 51), and we have recently reported reduced white matter volume and myelin loss in a subgroup of OVERTURE trail active arm participants who meet the inclusion criteria for detailed white matter and myelin content assessments (52). The current MRI findings of reduced brain volume loss are in accord with experimental results showing that 40 Hz sensory stimulation reduces neurodegeneration and brain atrophy via upregulation of cytoprotective proteins and a reduction of DNA damage (10).^.^ Prevention of cortical thinning in the occipital lobe could be due to several mechanisms revealed previously in experimental studies. Repeated application of 40 Hz sensory stimulation over a couple of weeks prevented depletion of presynaptic and postsynaptic markers, promoted dendritic spine maturation, and reduced neuronal loss in transgenic mice (10). Alzheimer’s Disease Neuroimaging Initiative (ADNI) studies have established a clear correlation between MRI-based brain atrophy and cognitive/functional abilities in both aging and subjects with AD spectrum (33, 34).

In summary, this study demonstrated safety, tolerability and adherence and preliminary evidence showing a reduction of cognitive and functional decline and brain atrophy following 6 months of treatment. It will be important to confirm the observed treatment effects of Cognito’s Evoked Gamma Therapy System (CogTx-001) on MMSE, ADCS-ADL and MRI outcomes in a randomized, sham-controlled pivotal clinical trial in mild to moderate AD patients. This trial will be paired with neuroimaging and established and exploratory AD biomarkers to provide mechanistic insights. The emerging safety profile and potential efficacy data suggests that this treatment modality could be considered as a stand-alone AD treatment or in combination with current or future pharmacotherapies.

## Data availability statement

The datasets presented in this article are not publicly available due to them containing information that could compromise research participant privacy/consent. Requests to access the datasets should be directed to the corresponding author.

## Ethics statement

The studies involving human participants were reviewed and approved by Advarra, a central Institutional Review Board, a full AAHRPP accredited research review service provider (https://www.advarra.com/). Advarra’s IRB Organization (IORG) Number is 0000635 and IRB Registration number is 00000971. The CIP was developed in accordance with the requirements set forth in the United States Code of Federal Regulation, 21 CFR 812 Investigational Device Exemptions, ISO 14155:2011 Clinical Investigations of Medical Devices for Human Subjects, the Medical Device Directive 93/42/EEC of the European Union, and the Declaration of Helsinki by the World Health Organization (as amended in 2008). The studies were conducted in accordance with the local legislation and institutional requirements. The participants provided their written informed consent to participate in this study.

## Author contributions

MH: Conceptualization, Formal analysis, Supervision, Writing – original draft, Writing – review & editing. AB: Conceptualization, Data curation, Writing – original draft. EH: Data curation, Investigation, Writing – original draft. MS: Data curation, Investigation, Writing – original draft. AK: Data curation, Investigation, Writing – original draft. CS: Conceptualization, Data curation, Formal analysis, Writing – original draft. VF: Data curation, Visualization, Writing – original draft. KK: Methodology, Supervision, Writing – original draft. JN-J: Data curation, Formal analysis, Writing – original draft. SH: Formal analysis, Validation, Writing – review & editing. BV: Conceptualization, Resources, Writing – review & editing. RK: Formal analysis, Writing – review & editing. JM: Conceptualization, Investigation, Supervision, Writing – review & editing. ZM: Conceptualization, Investigation, Methodology, Supervision, Writing – original draft.
