# Supplementary material for: Safety, tolerability, and efficacy estimate of evoked gamma oscillation in mild to moderate Alzheimer’s disease
Source: Front Neurol. 2024 Mar 6;15:1343588. doi: 10.3389/fneur.2024.1343588 (PMC10957179; doi:10.3389/fneur.2024.1343588)
Supplement: Supplementary file 1 [file Data_Sheet_1.pdf]

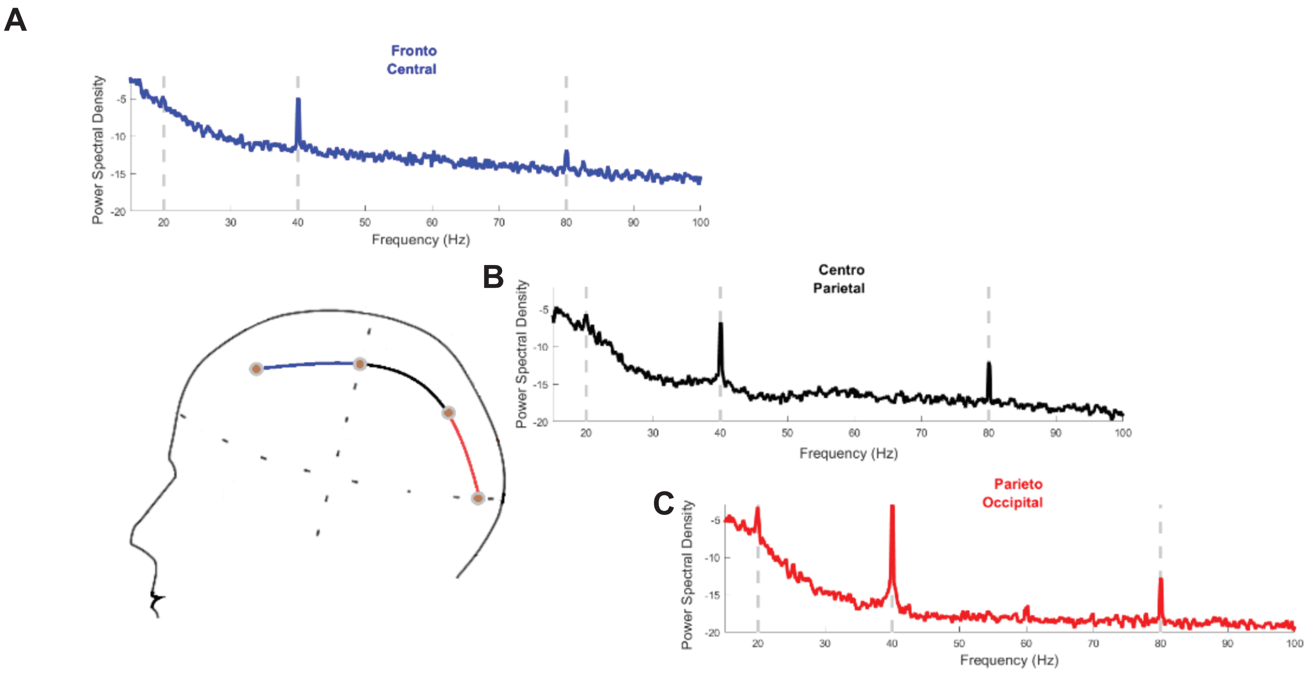

Supplementary Figure 1: Neuronal activity as observed from electroencephalogram (EEG) during 40Hz audio and visual sensory stimulation from a participant with Alzheimer's disease.

Neural response at 40Hz along with subharmonics and superharmonics of 40Hz can be observed in large cortical regions. Shown here are the neural activity from (A) Fronto-Central, (B) Centro-Parietal, and (C) Parieto-Occipital regions. In the Parieto-Occipital region the observed response is more pronounced.

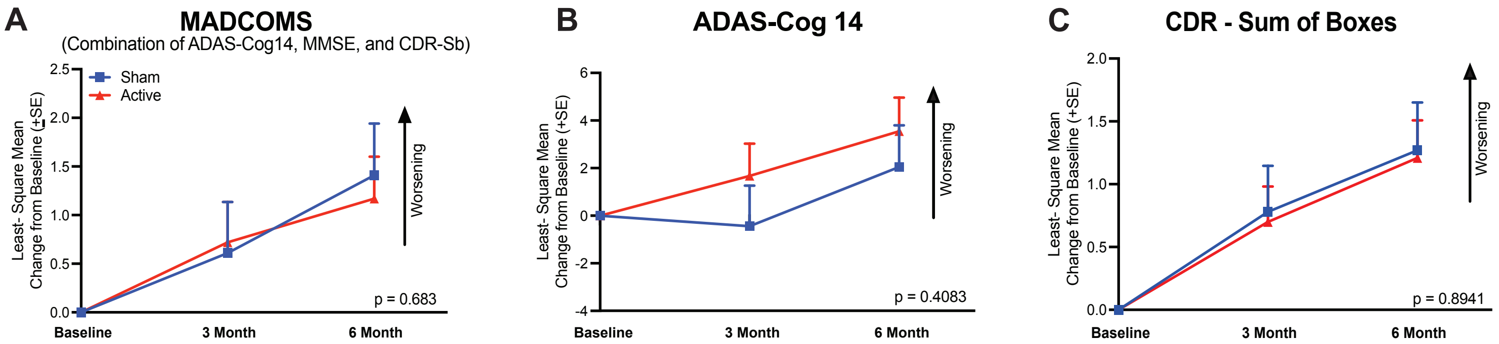

Supplementary Figure 2: Clinical outcome measures

Participants in the present trial did not show significant changes on traditional outcome measures. (A) MADCOMS (combination of ADAS-Cog14, MMSE, and CDR-sb) change from baseline scores in sham (n=26) vs active (n=43) participants (p=0.683). (B) ADAS-Cog14 change from baseline scores in sham (n=26) vs active (n=43) participants (p=0.408). (C)CDR – Sum of Boxes change from baseline scores in sham (n=27) vs active (n=43) in participants (p=0.894). All analyses were conducted using a linear mixed model with significance established as p<0.05. Arrows represent the direction of worsening.

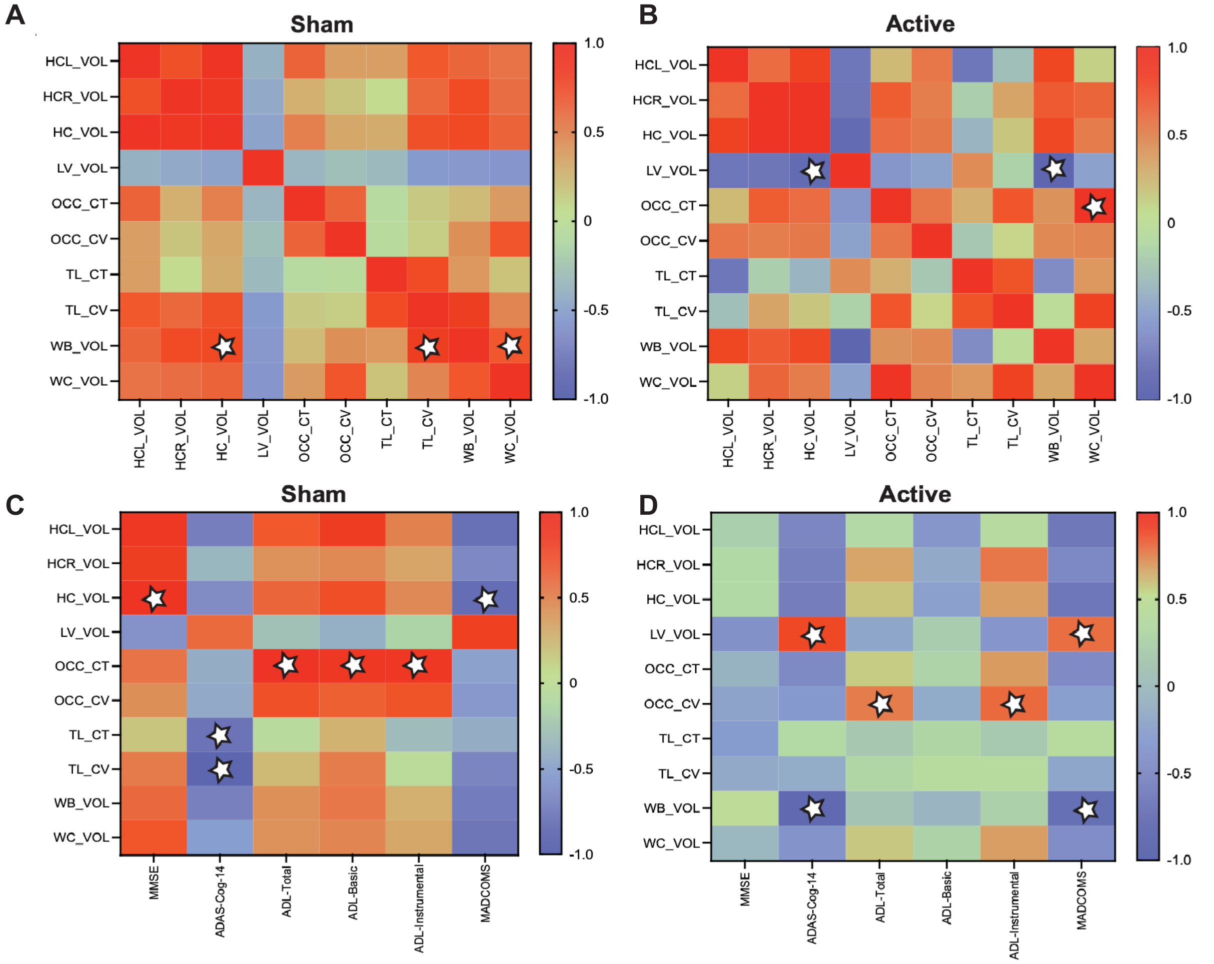

Supplementary Figure 3: Correlation matrixes between volumetric changes in different brain regions and correlation between volumetric changes and clinical outcomes

Correlation matrixes show correlation between volumetric changes in different brain regions in sham (A) and active (B) groups and correlation between volumetric changes and clinical outcomes in sham (C) and active (D) groups after 6 months of treatment. Matrixes A and B show r-value correlations in volumetric changes between 11 brain regions. The active group shows strong inverse correlations between volumetric changes of whole brain and lateral ventricle (r=-0.94, p=0.0001), lateral ventricle and hippocampus (r=-0.88, p=0.002), and positive correlation between occipital cortical thickness and whole brain cortical volume (r=0.97, p=0.00002). In sham group participants positive correlations were observed between whole brain and temporal cortical lobe volumes (r=0.85, p=0.01), whole brain and hippocampus (r=0.79, p=0.02), whole brain volume and whole brain cortical volume, brain regions that are known to be connected to AD disease progression. In addition, a correlation was found between changes in temporal lobe volume and temporal lobe cortical thickness in both sham (r=0.78, p=0.02) and active (r=0.74, p=0.02) groups. Stars represent relevant correlations discussed. (HCL_VOL: Left Hippocampus Volume; HCR_VOL: Right Hippocampus Volume; HC_VOL: Sum of the HCL and HCR Volume; LV_VOL: Lateral Ventricle Volume; OCC_CT Occipital Cortex Thickness; OCC_CV: Occipital cortical volume; TL_CT: Temporal Cortical Thickness; TL_CV: Temporal Cortical Volume; WB_VOL: Whole Brain Volume; WC_Vol: Whole Cortex Volume).

Matrixes C and D show r-value correlations in changes in volumetric values of 11 brain regions and select clinical outcomes (MMSE, ADASCog-14, ADCS-ADL Total, ADCS-ADL Basic, ADCS-ADL, Instrumental, and MADCOMS). In the sham group, volumetric changes of the hippocampus correlated with MADCOMS scores (r=-0.85, p=0.03) and MMSE (r=0.94, p=0.005), occipital cortical thickness with ADCS-ADL total (r=0.97, p=0.002) ADCS-ADL basic (r=0.91, p=0.01) and ADCS-ADL instrumental (r=0.92, p=0.01), and temporal lobe volume and cortical thickness with ADAS-Cog14 (r=-0.94, p=0.006; r=-0.81, p=0.05, respectively). In active arm participants, volumetric changes of the whole brain correlated with ADAS-Cog14 (r=-0.84, p=0.01) and MADCOMS (r=-0.77, p=0.02); lateral ventricle with ADAS-Cog14 (r=0.90, p=0.002) and MADCOMS (r=0.80, p=0.02); and occipital lobe with ADCS-ADL total (r=0.78, p=0.02) and ADCS-ADL instrumental (r=0.82, p=0.01). Stars represent relevant correlations discussed.

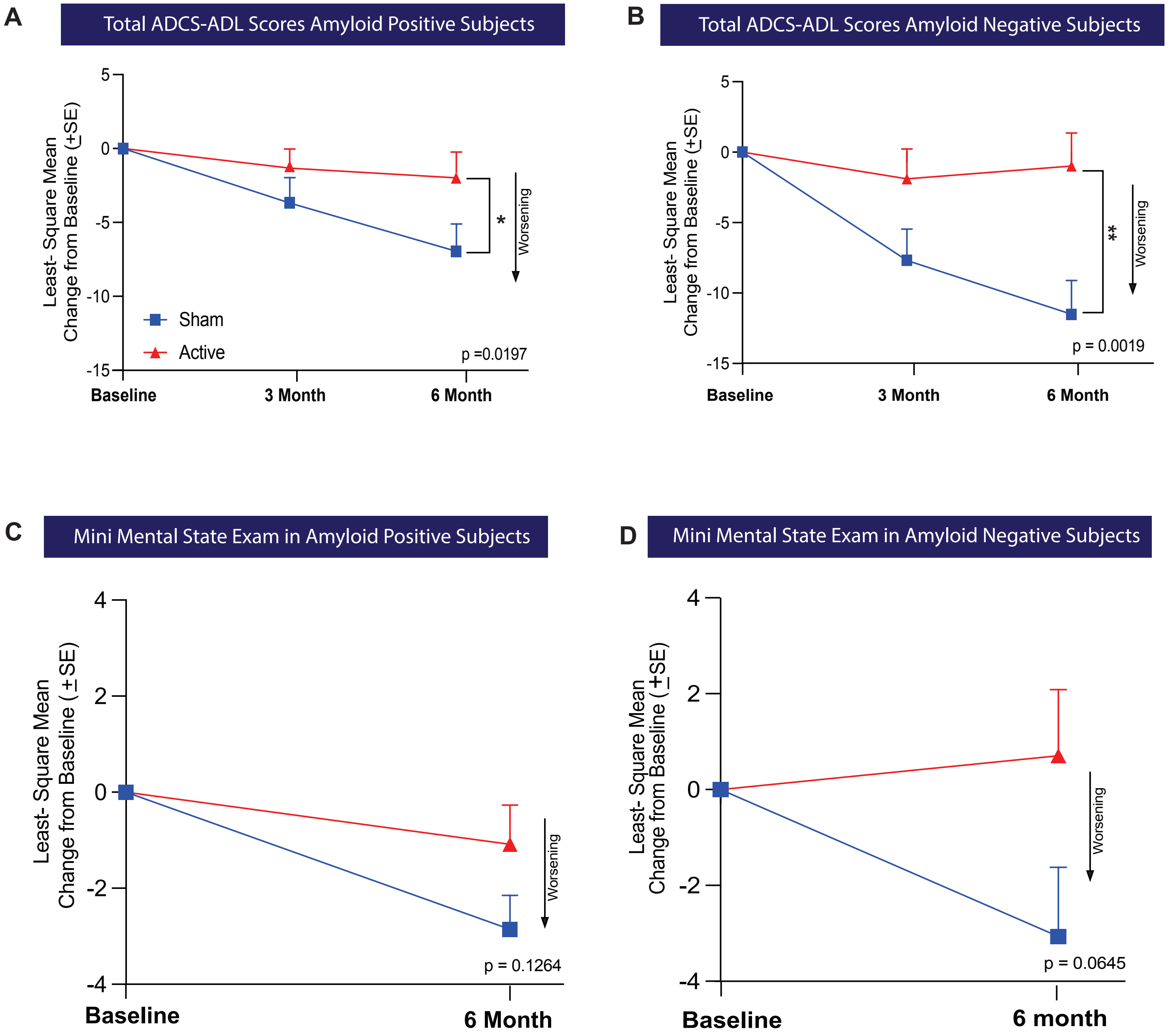

Supplementary Figure 4: Clinical outcome measures in amyloid positive and amyloid negative participant

Evoked gamma oscillation therapy is effective in both amyloid positive and amyloid negative participant. (A) ADCS-ADL total score change in amyloid positive participants in sham (n=17) vs active (n=26) participants at the end of the 6-month trial (p=0.020). (B) ADCS-ADL total score change in amyloid negative participants in sham (n=9) vs active (n=11) at the end of the 6-month trial (p=0.002). (C) MMSE total score change in amyloid positive participants in sham (n=18) vs active (n=32) at the end of the 6-month trial (p=0.126). (D) MMSE total score change in amyloid negative participants in sham (n=9) vs active (n=11) at the end of the 6-month trial (p=0.065). All analysis was conducted using a linear mixed model with significance established as p<0.05. *P<0.05, **P<0.005

Supplementary Table 1: Main Inclusion and Exclusion Criteria

Inclusion Criteria	
Subjects who met the following criteria were considered eligible to participate in the study:	
1	Was ≥ 50 years old at the time of consent.
2	Had a MMSE score ranging from 14-26, inclusive.
3	Had a diagnosis of a clinical syndrome of cognitive impairment consistent with prodromal AD, AD, or MCI due to AD per National Institute on Aging-Alzheimer's Association (NIA-AA) diagnostic criteria.
4	Could identify (or had already identified) a legally authorized representative who can verify study inclusion/exclusion criteria.
5	Had a reliable Lead Care Partner or informant (defined as an individual who knew subject well and had contact with subject for at least 10 hours each week or in investigator's opinion had sufficient knowledge of the subject's daily life to be a reliable informant and attend to the subject during treatment sessions) who also consented to study participation.
	NOTE: Up to 2 Alternate Care Partner(s) could be designated as well as a Lead Care Partner, provided each person also attended a clinic visit and signed the ICF and attended Initial Therapy or First At Home Therapy for training on device use from Field Support.
6	Showed adequate induced activity (entrainment) during baseline EEG.
7	Provided consent or assent (as appropriate) to participation in study assessments.

Exclusion Criteria	
Subjects who met any of the following criteria were not included in the study:	
1	Self- or caregiver reported concurrent profound hearing or visual impairment.
2	Self- or caregiver reported recent or clinically relevant history of seizure.
3	Active treatment or concurrent prescription for the purpose of preventing seizure with one or more anti-seizure/anti-epileptic.
4	Prior ischemic stroke, intracerebral hemorrhage, or subarachnoid bleed within the past 24 months, or that caused MRI exclusion criteria to be met.
5	Self- or caregiver reported usage of any new AD medication within the past 30 days, or concurrent/expected titration of dosage of any AD medications during the study period.
6	NOTE: AD medications and dosage had to be stable for 30 days at baseline neuropsychological assessments and initial therapy date. If doses were changed during screening, neuropsychological assessments had to be repeated.
7	Active treatment or concurrent prescription with memantine (Namenda™ , Namzaric™) within 30 days of baseline neuropsychological assessments and starting therapy.
8	Active treatment or concurrent prescription with medications that lower seizure threshold and pose a risk for participation in the study investigator's opinion.
9	Self- or caregiver reported of physician-diagnosis of a medical, neurological/neurodegenerative or psychiatric condition that in the opinion of the investigator could be contributing to the patient's cognitive impairment. This could include, but was not limited to, vitamin B12 deficiency, abnormal thyroid function, stroke or other cerebrovascular condition, traumatic brain injury, Lewy Body disease, Parkinson's disease, fronto-temporal dementia, schizophrenia, bipolar disorder, or uncontrolled major depressive disorder. Self- or caregiver report of current or historic migraines that in the investigator's opinion would potentially interfere with study compliance.
10	Self- or caregiver reported of physician-diagnosis of recent or clinically relevant major depressive disorder.
11	Self-report of clinically significant psychiatric illness or behavioral problem or use of psychiatric agent that could interfere with study completion, as determined by study physician.
12	Self- or caregiver reported alcohol or substance abuse within the past year.
13	Self- or caregiver reported concurrent enrollment in any anti-amyloid clinical trial within 30 days or 5 half-lives of the investigational intervention (whichever was longer) of the baseline neuropsychological assessments, baseline PET and starting therapy.
14	Subjects who, in the investigator's opinion would not comply with study procedures.
15	Subjects with active implantable neurological devices including deep brain stimulators (DBS) and/or non-MRI-safe implantable devices including pacemakers, implantable cardioversion defibrillators (ICDs), spinal cord stimulators, and non-MR compatible surgical implants.
16	Subject was pregnant, lactating, or of childbearing potential (i.e. women had to be 2 years post-menopausal, used 2 forms of birth control, or surgically sterile).
17	Previous amyloid imaging with 18F–AV-45 within the previous 3 months of baseline PET scan.
18	Concurrent or recent participation in any procedures involving radioactive agents such that the total radiation dose exposure to the subject in any given year would exceed the limits of annual and total dose commitment set forth in the US CFR Title 21 Section 361.1.
	Any of the following MRI findings:
	a. Prior macro-hemorrhages
	b. More than 4 micro-hemorrhages
	c. Any findings that prevent volumetric measurements necessary for PET analysis
19	Investigator opinion that it was not in the best interest of the subject to participate in the study.

Supplementary Table 2: ITT As treated and As Pre-Specified efficacy measure outcomes
iADRS, Integrated Alzheimer’s Disease Rating Scale; CDR-Global, Clinical Dementia Rating-Global; QoL-AD, Quality of Life in Alzheimer’s Disease (Self Report and Family Report)

Measure	ITT As Treated - Efficacy Measures						ITT As Prespecified - Efficacy Measures					
	Active		Sham		Active-Sham		Active		Sham		Active-Sham	
	N	Mean ± SE	N	Mean ± SE	Δ ± SE	P value	N	Mean ± SE	N	Mean ± SE	Δ ± SE	P value
**ADCOMS**	43	0.13 (0.034)	26	0.15 (0.041)	-0.02 (0.043)	0.644	42	0.12 (0.031)	27	0.16 (0.038)	-0.04 (0.041)	0.2898
**NPI**	36	0.09 (0.810)	26	-0.53 (0.964)	0.62 (1.031)	0.551	36	-0.20 (0.863)	26	-0.50 (1.001)	0.29 (1.024)	0.775
**ADAS-Cog 14**	43	3.55 (1.416)	26	2.05 (1.745)	1.51 (1.808)	0.408	42	3.28 (1.487)	27	2.27 (1.741)	1.01 (1.781)	0.572
**iADRS**	43	-5.19 (1.486)	25	-9.83 (2.098)	4.65 (2.686)	0.0906	42	-4.85 (1.503)	26	-10.1 (2.029)	5.26 (2.631)	0.052
**CDR-Global**	43	0.18 (0.056)	27	0.24 (0.080)	-0.05 (0.106)	0.607	42	0.16 (0.056)	28	0.27 (0.075)	-0.12 (0.101)	0.254
**CDR-Sum of Boxes**	43	1.21 (0.298)	27	1.27 (0.381)	-0.06 (0.434)	0.894	42	1.07 (0.263)	28	1.35 (0.334)	-0.28 (0.412)	0.503
**Care Partner Burden**	43	-0.15 (1.835)	27	1.96 (2.153)	-2.11 (2.126)	0.321	42	-0.18 (1.878)	28	1.90 (2.137)	-2.07 (2.112)	0.327
**QoL-AD (Self Report)**	43	0.67 (0.637)	27	0.78 (0.868)	-0.11 (1.115)	0.919	42	0.67 (0.646)	28	0.78 (0.844)	-0.11 (1.096)	0.919
**QoL-AD (Family Report)**	43	-0.12 (0.578)	27	-1.22 (0.775)	1.10 (1.001)	0.272	42	-0.12 (0.585)	28	-1.15 (0.753)	1.04 (0.981)	0.292
**Temporal Cortical Volume**	31	-1.77 (0.263)	15	-1.37 (0.417)	-0.40 (0.510)	0.441	30	-1.76 (0.266)	16	-1.46 (0.396)	-0.30 (0.489)	0.545
**Temporal Cortical Thickness**	31	-0.03 (0.009)	15	-0.02 (0.015)	-0.01 (0.018)	0.573	30	-0.03 (0.010)	16	-0.02 (0.014)	-0.01 (0.018)	0.524
**Right Hippocampus**	27	-0.01 (0.031)	12	-0.03 (0.034)	0.02 (0.028)	0.568	27	-0.01 (0.031)	12	-0.03 (0.034)	0.02 (0.028)	0.568
**Left Hippocampus**	28	-0.03 (0.010)	14	-0.04 (0.014)	0.01 (0.018)	0.679	27	-0.03 (0.011)	15	-0.04 (0.013)	0.01 (0.017)	0.548
**Total Hippocampus**	24	-0.06 (0.019)	10	-0.08 (0.028)	0.01 (0.033)	0.677	24	-0.06 (0.019)	10	-0.08 (0.028)	0.01 (0.033)	0.677
**Whole Cortex Volume**	32	-6.79 (1.671)	16	-9.51 (2.509)	2.71 (3.126)	0.392	31	-7.25 (1.703)	17	-8.61 (2.421)	1.36 (3.049)	0.658

Supplementary Table 3: Efficacy measure segmentation between ITT as treated amyloid beta positive and negative participants.

	AB-						AB+					
	Active		Sham		Active-Sham		Active		Sham		Active-Sham	
	N	Mean + SE	N	Mean + SE	Δ + SE	P value	N	Mean + SE	N	Mean + SE	Δ + SE	P value
**ADCS-ADL (Total)**	11	-0.98 (2.34 2)	9	-11.51 (2.408)	10.53 (3.349)	0.002	26	-1.99 (1.355)	17	-6.95 (1.846)	4.96 (2.113)	0.02
**ADCS-ADL (Instrumental)**	11	-0.57 (1.819)	9	-9.34 (1.859)	8.77 (2.590)	0.001	32	-1.66 (1.029)	17	-5.76 (1.393)	4.10 (1.580)	0.01
**ADCS-ADL (Basic)**	11	-0.32 (0.700)	9	-2.68 (0.733)	2.36 (1.040)	0.024	32	-0.36 (0.366)	18	-1.63 (0.519)	1.27 (0.636)	0.046
**MMSE**	11	0.71 (1.379)	9	-3.06 (1.443)	3.77 (1.994)	0.065	32	-1.09 (0.819)	18	-2.85 (1.049)	1.77 (1.135)	0.126
**Whole Brain Volume**	7	1.23 (5.946)	5	-20.92 (5.909)	22.148 (8.675)	0.015	29	-6.56 (2.490)	15	-16.57 (3.620)	10.01 (4.422)	0.03
**Whole Cortex Volume**	7	-1.19 (4.207)	4	-12.498 (4.729)	11.31 (6.628)	0.099	25	-7.75 (1.822)	12	-9.03 (2.919)	1.28 (3.487)	0.716
**Lateral Ventricle**	6	1.74 (1.042)	4	5.39 (1.144)	-3.65 (1.602)	0.029	28	3.10 (0.423)	15	3.12 (0.635)	-0.02 (0.779)	0.982
**Occipital Cortical Volume**	4	0.28 (0.591)	4	-1.18 (0.438)	1.46 (0.735)	0.059	23	-0.21 (0.195)	10	-0.88 (0.307)	0.67 (0.382)	0.091
